# Mitochondrial Hsp90s suppress calcium-mediated stress signals propagating from mitochondria to the ER in cancer cells

**DOI:** 10.1186/1476-4598-13-148

**Published:** 2014-06-12

**Authors:** Hye-Kyung Park, Ji-Eun Lee, Jaehwa Lim, Byoung Heon Kang

**Affiliations:** 1 Department of Biological Sciences, School of Life Sciences, Ulsan National Institute of Science and Technology (UNIST), 50 UNIST St., Ulsan 689-798, South Korea

**Keywords:** Mitochondrial Hsp90s, Mitochondrial permeability transition pore, Ryanodine receptor, Calcium signaling, Combination cancer therapy

## Abstract

**Background:**

Resistance to cell death in the presence of stressful stimuli is one of the hallmarks of cancer cells acquired during multistep tumorigenesis, and knowledge of the molecular mechanism of stress adaptation can be exploited to develop cancer-selective therapeutics. Mitochondria and the endoplasmic reticulum (ER) are physically interconnected organelles that can sense and exchange various stress signals. Although there have been many studies on stress propagation from the ER to mitochondria, reverse stress signals originating from mitochondria have not been well reported.

**Methods:**

After inactivation of the proteins by pharmacologic and genetic methods, the signal pathways were analyzed by fluorescence microscopy, flow cytometry, MTT assay, and western blotting. A mouse xenograft model was used to examine synergistic anticancer activity and the action mechanism of drugs *in vivo*.

**Results:**

We show in this study that mitochondrial heat shock protein 90 (Hsp90) suppresses mitochondria-initiated calcium-mediated stress signals propagating into the ER in cancer cells. Mitochondrial Hsp90 inhibition triggers the calcium signal by opening the mitochondrial permeability transition pore and, in turn, the ER ryanodine receptor, via calcium-induced calcium release. Subsequent depletion of ER calcium activates unfolded protein responses in the ER lumen, thereby increasing the expression of a pro-apoptotic transcription factor, CEBP homologous protein (CHOP). Combined treatment with the ER stressor thapsigargin and the mitochondrial Hsp90 inhibitor gamitrinib augmented interorganelle stress signaling by elevating CHOP expression, and showed synergistic cytotoxic activity exclusively in cancer cells *in vitro* and *in vivo*.

**Conclusions:**

Collectively, mitochondrial Hsp90s confer cell death resistance to cancer cells by suppressing the mitochondria-initiated calcium-mediated interorganelle stress response.

## Background

Molecular chaperones assist in the correct folding and conformational changes of their substrates, called client proteins, and minimize their misfolding and aggregation [1]. Heat shock protein 90 (Hsp90) is an ATP-dependent molecular chaperone regulating the stability and functions of client proteins that are often involved in signal transduction during malignant transformation and progression [2,3]. Organelle-resident Hsp90 family proteins are present in mitochondria and the endoplasmic reticulum (ER), where they control protein homeostasis [4-6]. Hsp90 and its mitochondrial homolog, tumor necrosis factor receptor-associated protein 1 (TRAP1), are abundant in the mitochondria of many cancer cells [7-10], and their regulation, client proteins, and cellular functions are quite different from the cytoplasmic Hsp90 pool [4,11]. Mitochondrial Hsp90s are involved in tumor progression, cytoprotection, and multidrug resistance, by reprogramming cancer cell metabolism [12-16] and maintaining mitochondrial membrane integrity [7,17,18].

Mitochondria integrate lethal and vital signals emanating from various cellular compartments to cause cell death through inner and outer membrane permeabilization [19]. Though the molecular mechanism is not fully elucidated, cyclophilin D (Cyp-D) is believed to regulate the permeability transition pore (PTP) in the mitochondrial inner membrane [20-24]. Cancer cells elevate mitochondrial Hsp90 expression, which suppresses Cyp-D function to inhibit the deadly increase of membrane permeability in the organelle [7]. PTP opening upon Cyp-D activation increases mitochondrial inner membrane permeability toward small molecules (<1,500 Da), resulting in loss of mitochondrial membrane potential (Δ*Ψ*m), discharge of matrix calcium stores, and swelling and rupture of the mitochondrial outer membrane [19,25].

Calcium, a ubiquitous second messenger, is involved in a broad variety of physiological events *via* its interaction with effectors responsible for calcium-dependent processes [26]. The ER and mitochondria are the major intracellular calcium stores, regulating calcium homeostasis and signaling [27,28]. They have a largely interconnected architecture with numerous contacts, which facilitates inter-organelle calcium transport by generating calcium hotspots proximal to open calcium channels [29-31]. Both the ER and mitochondria contain calcium-triggered calcium release channels that can activate each other via positive feedback, including ryanodine receptors (RyRs) and inositol 1,4,5-trisphosphate receptors (IP_3_Rs) [19,32]. There is a growing consensus that ER-mitochondria calcium crosstalk can coordinate signaling for metabolism and cell death between the organelles [28].

Although calcium signaling has been intensively studied, reports of “mitochondria-initiated” calcium crosstalk between mitochondria and the ER are scarce. Here, we demonstrate a novel function of mitochondrial Hsp90s that confers resistance to cancer cell death by inhibiting the propagation of mitochondrial-origin calcium signals to the ER.

## Results

### Mitochondrial Hsp90s modulate the mitochondrial calcium store

**Figure 1:**
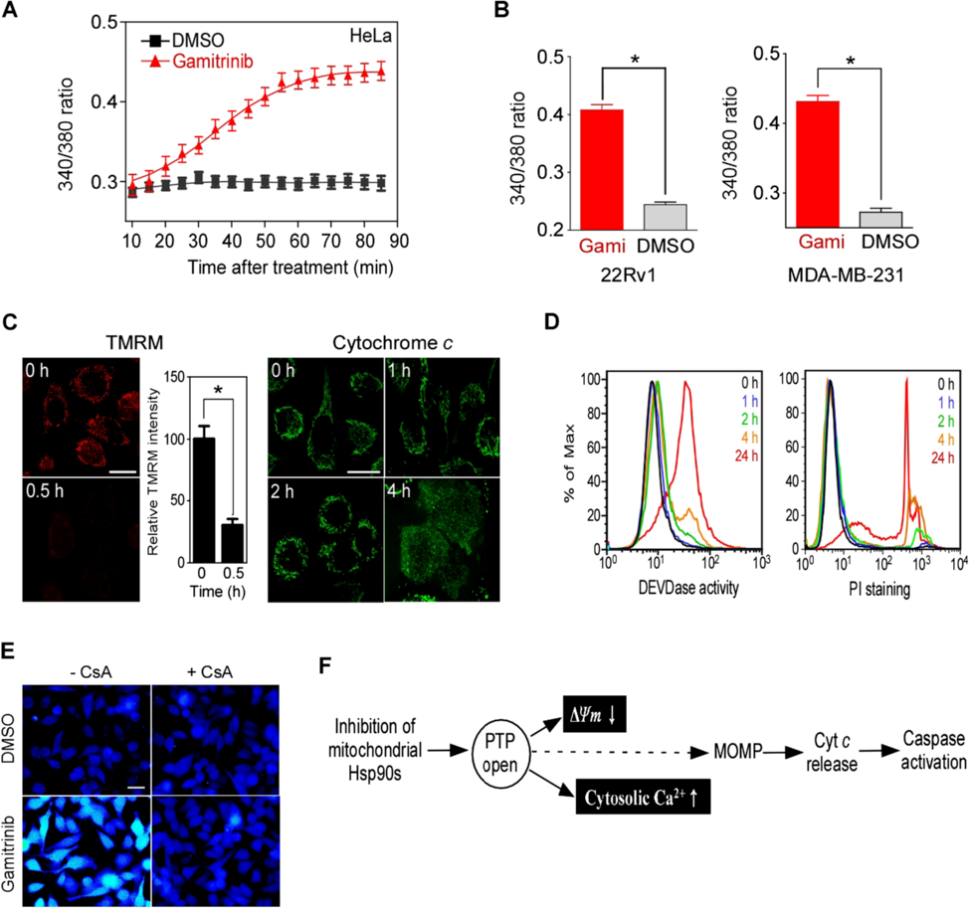
**Mitochondrial Hsp90s modulate the mitochondrial calcium store. (A)** Time course of cytosolic calcium increase. The ratio of the emission fluorescence intensities at 340 and 380 nm excitation of Fura-2 labeled HeLa cells in calcium-free medium was measured after 30 μM gamitrinib treatment as described in Materials and Methods. **(B)** Increase of cytosolic calcium in 22Rv1 and MDA-MB-231 cells. Fura-2 fluorescence ratio after 30 μM gamitrinib (Gami) treatment for 1 hour was calculated. Data are the mean ± SEM of duplicated experiments and collected from 40 regions of interest (ROIs). **(C)** Mitochondrial membrane permeabilization. TMRM-loaded HeLa cells were imaged to measure mitochondrial membrane potential depolarization (Δ*Ψm*) (left); alternatively, cytochrome *c* redistribution was analyzed (right) at the indicated times after 30 μM gamitrinib treatment as previously described [35]. White bar, 20 μm. **(D)** Caspase activation and cell death induction. After 30 μM gamitrinib treatment, HeLa cells were labeled with FITC-DEVD-fmk (left, DEVDase activity) or propidium iodide (right, PI staining) and analyzed by flow cytometry at the selected time points. **(E)** Cyclosporin A (CsA) blocks cytosolic calcium increase. Cytosolic calcium changes in Fura-2-labeled HeLa cells treated for 1 hour with 5 μM CsA and/or 30 μM gamitrinib were analyzed. Bar, 50 μm. **(F)** Summary of sequential events following mitochondrial Hsp90 inhibition. PTP opening is directly linked with the loss of Δ*Ψm* and increase of cytosolic calcium. The calcium flux occurs prior to mitochondrial outer membrane permeabilization (MOMP) and cytochrome *c* release. *, *p* < 0.0001.

To investigate whether mitochondrial Hsp90s modulate mitochondrial calcium stores, we used the mitochondria-targeted Hsp90 inhibitor gamitrinib, a conjugated of triphenylphosphonium (a mitochondria-targeting moiety) and geldanamycin (an Hsp90 inhibitor) [33,34]. A cytotoxic dose (30 μM) of gamitrinib dramatically increased the intracellular calcium concentration within an hour in human cervical (HeLa), prostate (22Rv1), and breast (MDA-MB-231) cancer cell lines in calcium-free medium (Figure 1A and B). A non-targeted Hsp90 inhibitor, 17-allylamino-17-demethoxygeldanamycin (17AAG), did not increase cytosolic calcium (Additional file 1: Figure S1A), consistent with a previous report that gamitrinib is specific to mitochondrial Hsp90 without affecting cytosolic Hsp90 function [33]. After gamitrinib treatment, PTP opening and loss of mitochondrial membrane potential (Δ*Ψm*) occurred within 30 minutes (Figure 1C, TMRM staining), whereas cytochrome *c* release, caspase activation, and cell death were not prominent until after 2 hours (Figure 1C, cytochrome *c* staining; Figure 1D), suggesting that calcium flux concurs with PTP opening, prior to mitochondrial outer membrane permeabilization (MOMP). Consistently, cytosolic calcium elevation was inhibited by cyclosporin A (CsA) (Figure 1E), a potent Cyp-D inhibitor, blocking PTP opening [19]. Thus, mitochondrial Hsp90 inhibition immediately induces PTP opening, loss of Δ*Ψm*, and discharge of the calcium stored in the mitochondrial matrix. Thereafter, a cascade of MOMP, cytochrome *c* release, and caspase activation ensues (Figure 1F).

### Mitochondrial calcium release results in depletion of ER calcium

**Figure 2:**
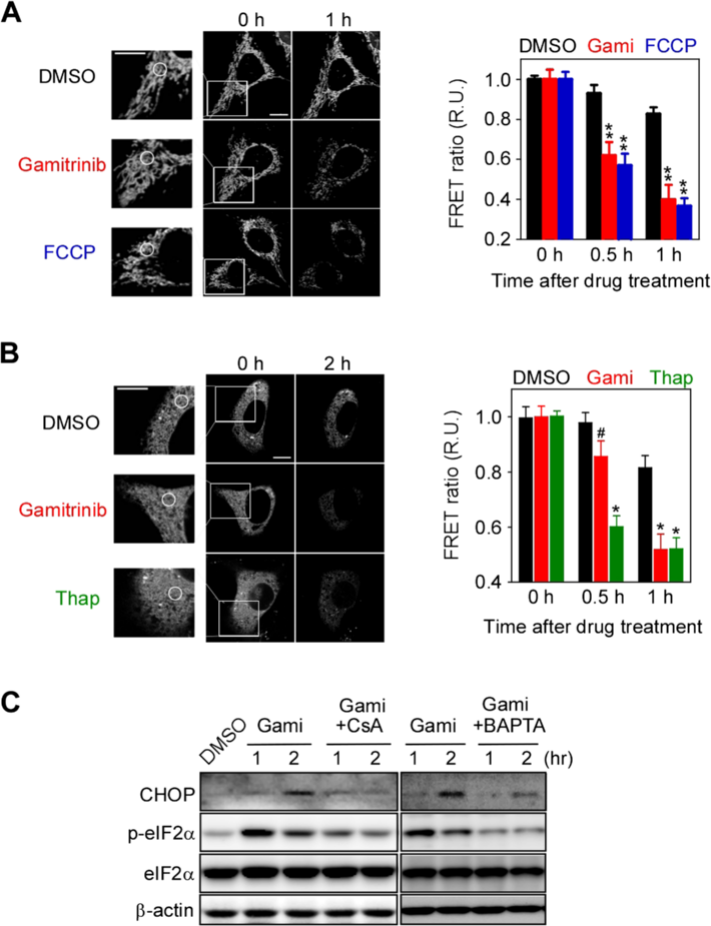
**Inhibition of mitochondrial Hsp90s depletes stored calcium in both mitochondria and the ER. (A)** Mitochondrial calcium depletion. After 30 μM gamitrinib and 10 μM FCCP treatment, confocal FRET images of mtCameleon-expressing HeLa cells were reconstructed from their emission fluorescence ratios at 535/480 nm with excitation at 440 nm (left). FRET ratios at the indicated time intervals were averaged and plotted (right). **(B)** ER calcium depletion. FRET images of HeLa cells transiently expressing D1ER were acquired at the indicated time points after gamitrinib treatment (left) and analyzed to plot the FRET ratio (right). Selected ROIs are indicated as white circles. Bar, 10 μm. Data in **(A)** and **(B)** are mean ± SEM collected from 30 ROIs. R.U., relative units. **(C)** CHOP induction and eIF2α phosphorylation. HeLa cells were treated with 30 μM gamitrinib, 5 μM CsA, and 10 μM BAPTA as indicated and analyzed by western blotting. #, not significant; *, *p* < 0.001; **, *p* < 0.0001.

The PTP opening has been shown to immediately discharge calcium stored in the mitochondria [36]; however, after mitochondrial Hsp90 inhibition in this study, calcium release continued even after a significant drop in Δ*Ψm* (Figure 1A and C), suggestive of additional sources of calcium flux. We postulated that the primary calcium-storing organelle, the ER, contributes to the cytosolic calcium increase after gamitrinib treatment. To prove this, we directly measured calcium depletion using the calcium sensor protein, Cameleon, targeted to mitochondria and the ER (mtCameleon and D1ER, respectively) [37]. Gamitrinib treatment resulted in FRET signal loss in both mtCameleon- and D1ER-transfected HeLa cells, comparable to that seen with FCCP or Thap treatment (Figure 2A and B), clearly indicating calcium depletion in the ER as well as in mitochondria. Consistent with previous reports [33], gamitrinib has no effect on the Δ*Ψm* of a normal MCF10A breast cell (Additional file 1: Figure S1B and C), and the non-targeted Hsp90 inhibitor 17AAG did not affect the mtCameleon FRET signal (Additional file 1: Figure S1D).

### Calcium depletion in the ER evokes the unfolded protein response and induces CHOP activation

Gamitrinib has been reported to trigger the unfolded protein response in mitochondria, and, through unknown mechanisms, to subsequently activate CHOP, the pro-apoptotic transcription factor often induced during unfolded protein responses in the ER (UPR^ER^) [4,38-40]. siRNA knockdown of the mitochondrial Hsp90 homolog TRAP1 results in spliced XBP1 mRNA production and eukaryotic translation initiation factor 2α (eIF2α) phosphorylation (Additional file 1: Figure S2A and B), suggesting activation of UPR^ER^ sensor proteins such as inositol-requiring protein 1α (IRE1α) and PKR-like ER kinase [41,42]. Consistently, pharmacological inactivation of mitochondrial Hsp90s by gamitrinib also triggered eIF2α phosphorylation and XBP1 mRNA splicing (Figure 2C; Additional file 1: Figure S2C). In addition to UPR^ER^ sensor protein activation, CHOP induction was clearly seen after both pharmacological and genetic inhibition of mitochondrial chaperones (Figure 2C; Additional file 1: Figure S2D). To investigate the critical involvement of mitochondrial calcium discharge through the PTP for the ER stress response, gamitrinib was administered in the presence or absence of the PTP inhibitor CsA and the calcium chelator BAPTA. Both substances compromised UPR^ER^ induction, resulting in a dramatic reduction in eIF2α phosphorylation and CHOP expression (Figure 2C).

### Ryanodine receptors mediate mitochondrial calcium-induced calcium depletion in the ER

**Figure 3:**
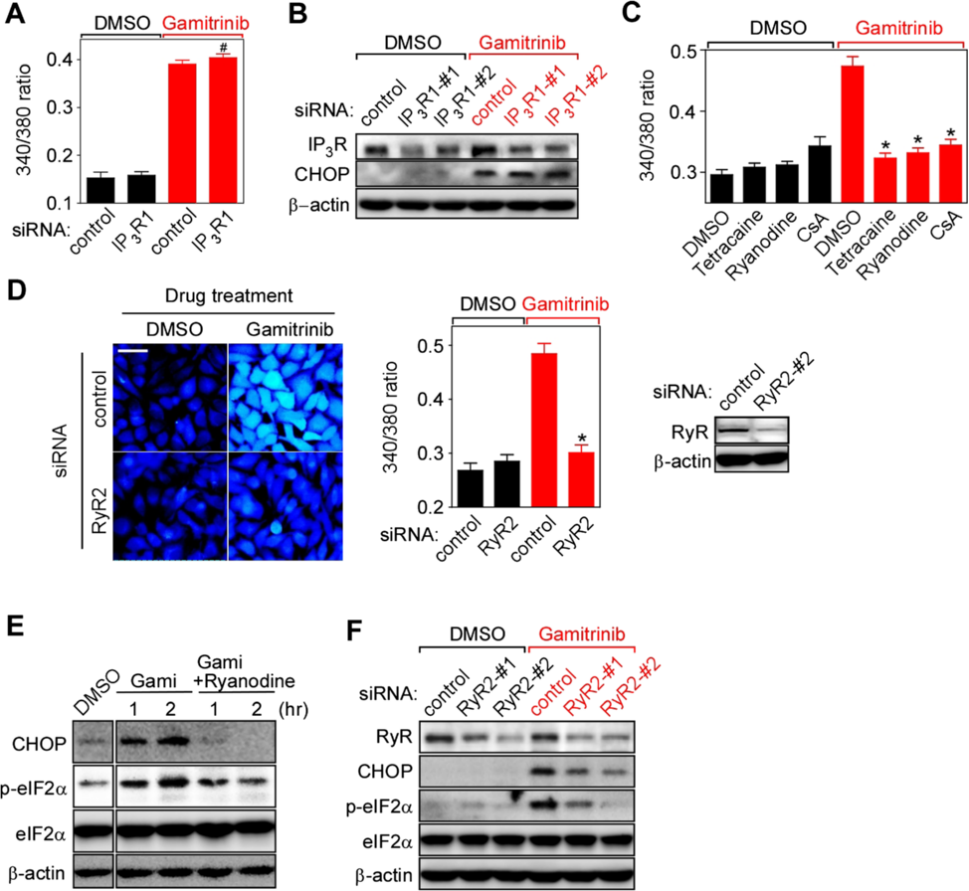
**Ryanodine receptor (RyR)-mediated cytosolic calcium elevation. (A)** IP_3_R silencing. After IP_3_R siRNA treatment, Fura-2 labeled HeLa cells were treated with 30 μM gamitrinib for 1 hour. The fluorescence ratio (340/380) was plotted. The data are mean ± SEM collected from 30 ROIs in two independent experiments. **(B)** IP_3_R knockdown effect on CHOP expression. Control or IP_3_R1 siRNA-transfected HeLa cells were incubated with or without 30 μM gamitrinib for 2 hours. Cell extracts were analyzed by western blotting. **(C)** RyR inhibitors compromise cytosolic calcium increase. Fura-2 labeled HeLa cells were treated with 30 μM gamitrinib for an hour in the presence or absence of 300 μM tetracaine, 100 μM ryanodine, and 5 μM CsA, and emission fluorescence intensity ratios (340/380 nm excitation) were measured. Data are mean ± SEM calculated from 40 ROIs in two independent experiments. **(D)** Fura-2 imaging and RyR2 silencing. Control or RyR2-#2 siRNA-treated HeLa cells were labeled with Fura-2 and imaged after 30 μM gamitrinib treatment for an hour (left). The fluorescence ratio (340/380) was plotted (middle). Knockdown efficiency of RyR2-#2 siRNA by western blotting (right). The data are mean ± SEM collected from 30 ROIs in two independent experiments. Bar, 50 μm. **(E)** Inhibition of CHOP induction by RyR inactivation. HeLa cells were treated with 30 μM gamitrinib in the presence or absence of 100 μM ryanodine. Cell extracts were analyzed by western blotting. **(F)** RyR2 silencing and CHOP expression. HeLa cells were treated with two different RyR2 siRNAs, incubated with 30 μM gamitrinib, for 2 hours and analyzed by western blotting. #, not significant; *, *p* < 0.0001.

IP_3_Rs and RyRs are ER membrane channels responsible for calcium release from the organelle [26]. Silencing IP_3_R1, the major isoform in HeLa cells [43] (Additional file 1: Figure S3A), did not affect the elevation of cytosolic calcium and the induction of CHOP after gamitrinib treatment (Figure 3A and B), but was enough to compromise lysophosphatidic acid-induced ER calcium release in calcium-free medium (Additional file 1: Figure S3B). By contrast, specific RyR inhibitors such as ryanodine (100 μM) and tetracaine (300 μM) [44] strongly inhibited gamitrinib-induced ER calcium release, similar to the PTP inhibitor CsA (Figure 3C). Genetic knockdown of RyR2, the dominant RyR isoform in HeLa cells [45-47], also blocked gamitrinib-induced cytoplasmic calcium increase (Figure 3D). Consistently, ryanodine and RyR2-specific siRNAs inhibited eIF2α phosphorylation and the subsequent CHOP induction (Figure 3E and F). Collectively, our data suggest that RyR, not IP_3_R, is the ER sensor that propagates the signal initiated by discharged calcium from mitochondria in cancer cells.

### Mitochondria-initiated calcium signaling plays an important role in setting up the cell death threshold

**Figure 4:**
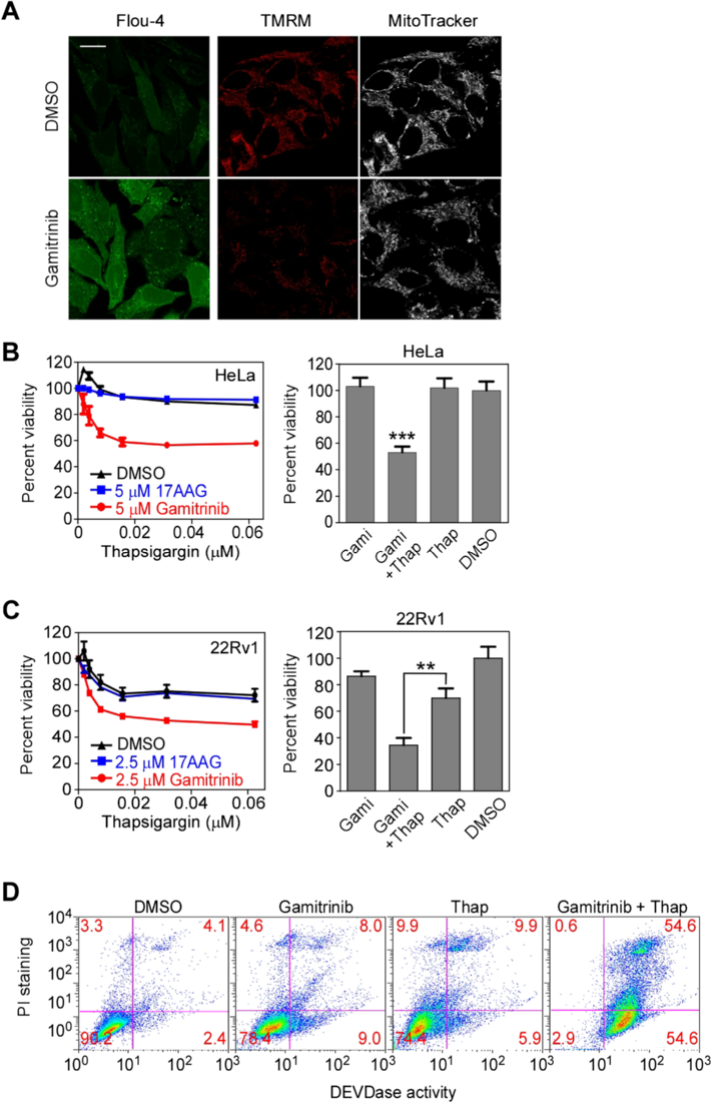
**Inhibition of mitochondrial Hsp90s sensitizes HeLa cells toward thapsigargin. (A)** Cytoplasmic calcium and mitochondrial membrane potential by suboptimal dose of gamitrinib. Fluo-4 or TMRM/MitoTracker-labeled HeLa cells were incubated with 5 μM gamitrinib for 24 hours and analyzed by confocal microscope. Bar, 20 μm. **(B)** Combination effect in HeLa. HeLa cells were treated with various concentrations of Thap in the presence of 5 μM of either 17AAG or gamitrinib, and analyzed by MTT assay (left). Alternatively, HeLa cells were treated with 5 μM gamitrinib and/or 0.06 μM Thap for 24 hours and analyzed by the MTT assay. ***, *p* < 0.0001. **(C)** Combination effect in 22Rv1. 22Rv1 cells were treated with various concentrations of thapsigargin in the presence of 2.5 μM of either 17AAG or gamitrinib for 24 hours, and analyzed by the MTT assay (left). Alternatively, 22Rv1 cells were treated with 2.5 μM gamitrinib (Gami) and 0.06 μM Thap as indicated for 24 hours and analyzed by the MTT assay. **, *p =* 0.0006. **(D)** Combination treatment induces apoptosis. HeLa cells were treated with 10 μM gamitrinib and 0.5 μM Thap as indicated, labeled with FITC-DEVD-fmk and propidium iodide, and analyzed by flow cytometry. **(B)** and **(C)** represent mean ± SEM from three independent experiments.

Impaired mitochondrial function [16] (Figure 4A, TMRM staining) and slightly elevated cytoplasmic calcium (Figure 4A, Fluo-4 staining) were frequently found in gamitrinib-treated cells, even at non-toxic dose of the drug. Therefore, we hypothesized that calcium-mediated stress propagation can render cells sensitive to additional stresses, *i.e.* lowering the cell death threshold. A representative UPR^ER^ inducer, Thap, was combined with gamitrinib to test this hypothesis. Gamitrinib sensitized cancer cells to Thap treatment at various concentrations, while the non-targeted Hsp90 inhibitor 17AAG did not (Figure 4B and C). Consistent with pharmacological data, TRAP1 knockdown also sensitized cancer cells to Thap treatment (Additional file 1: Figure S4). The combination of gamitrinib and Thap synergistically induced apoptotic cell death, causing a dramatic increase in caspase activity (Figure 4D).

### Gamitrinib and Thap together elevate CHOP expression in an RyR-dependent manner

**Figure 5:**
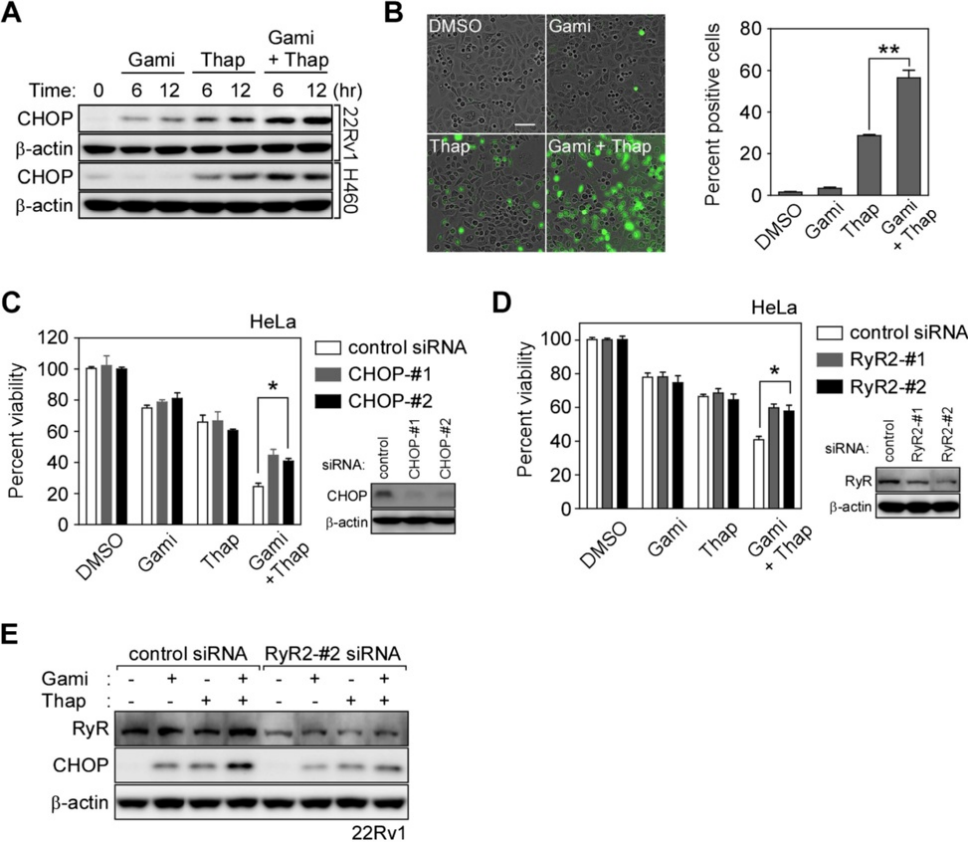
**Gamitrinib and Thap combination treatment elevates CHOP expression. (A)** Synergistic increase in CHOP induction. 22Rv1 and H460 cells were treated with 5 μM gamitrinib and 0.06 μM Thap as indicated and analyzed by western blotting. **(B)** CHOP reporter assay. PC3 cells stably transfected with a *CHOP::GFP* reporter plasmid [50] were incubated with 2.5 μM gamitrinib and/or 0.02 μM Thap as indicated and analyzed as described in Materials and Methods (left). Cells with more than twice the background fluorescence intensity were considered as positive cells (right). Bar, 100 μm. Mean ± SEM. **, *p* = 0.0036. **(C)** Silencing CHOP expression. Control or CHOP siRNA-transfected HeLa cells were treated with 0.06 μM Thap and 5 μM gamitrinib for 24 hours, and analyzed by MTT assay. Knockdown efficiency analyzed by western blotting (bottom right). Mean ± SEM. *, *p* < 0.05. **(D)** Silencing RyR expression. Control or RyR siRNA-treated HeLa cells were incubated with 0.06 μM Thap and 5 μM gamitrinib for 24 hours, and analyzed by MTT assay. Knockdown efficiency analyzed by western blotting (bottom right). Mean ± SEM. *, *p* < 0.05. **(E)** Knockdown of RyR2 by siRNA. Control or RyR2 siRNA-transfected 22Rv1 cells were incubated with 2.5 μM gamitrinib and/or 0.06 μM Thap as indicated and analyzed by western blotting.

Proapoptotic CHOP expression induced by combination treatment with gamitrinib and Thap was faster and higher compared to single-agent treatment (Figure 5A). A cell-based reporter assay also showed elevated CHOP transcription activity following combination treatment (Figure 5B). siRNA-mediated knockdown of either CHOP or RyR significantly suppressed this increased cytotoxic activity, but did not affect the toxicity seen with single-agent treatment (Figure 5C and D), suggesting important roles of RyR and CHOP in the drug combination effect. Silencing RyR compromised CHOP induction by the drug combination but not by single-agent treatment (Figure 5E), further confirming that RyR opening is an essential upstream event in the stress response elevating CHOP expression. CHOP-dependent death receptor 5 (DR5) expression [48] has been reported before, but was not involved in the drug combination, considering marginal elevation of DR5 expression and no activation of caspase-8 (Additional file 1: Figure S5A) [49]. Neither did reactive oxygen species (ROS) scavengers affect the increase in cytoplasmic calcium and CHOP induction (Additional file 1: Figure S5B and C). Collectively, our data argue that gamitrinib lowers the cellular threshold against ER stresses by increasing CHOP expression in an RyR-dependent manner.

### Combined synergistic anticancer activities *in vivo*

**Figure 6:**
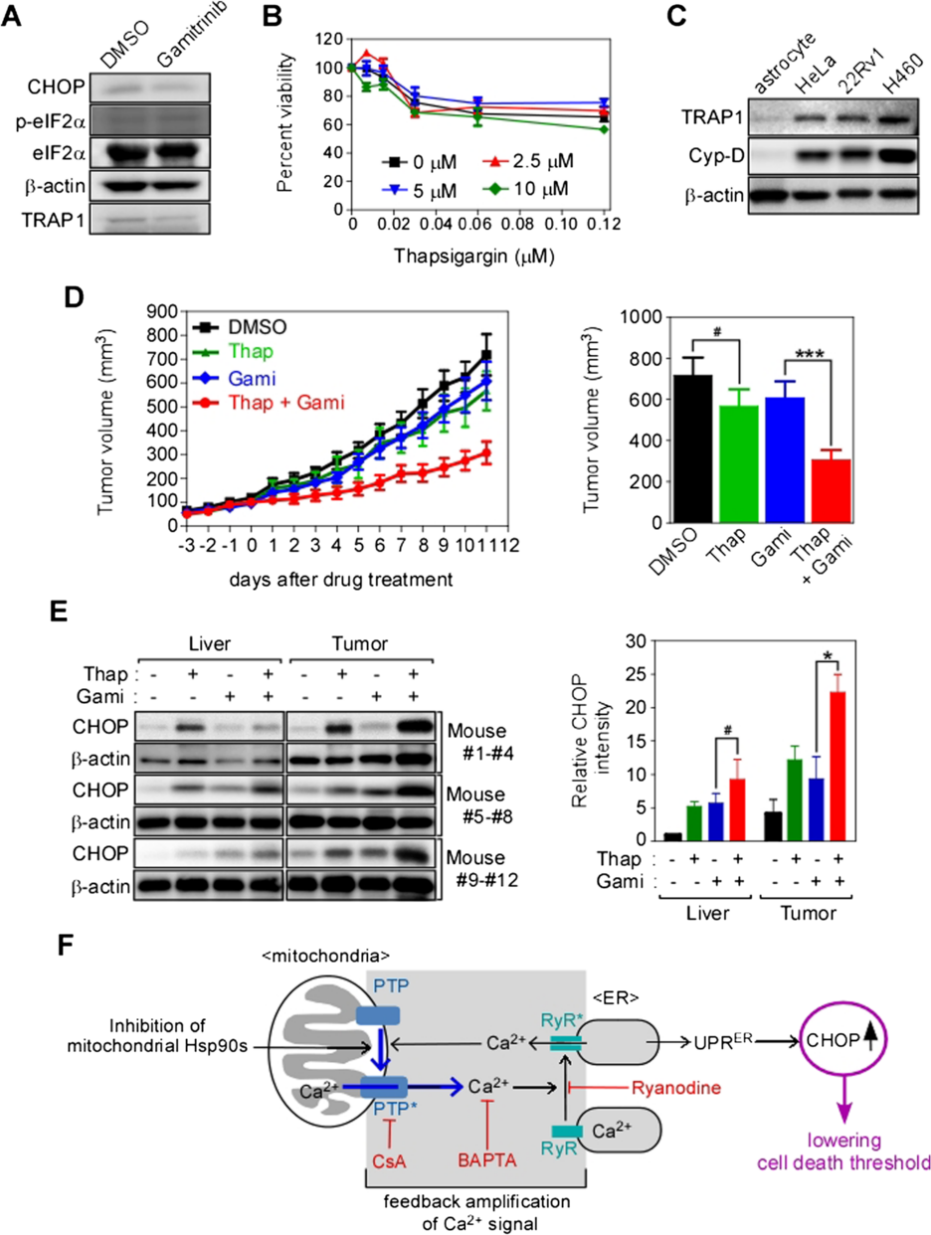
**Synergistic cancer-specific cytotoxicity *in vivo*. (A)** CHOP induction in astrocytes. Astrocytes treated with 30 μM gamitrinib for 2 hours were analyzed by western blotting. **(B)** Thap in combination with gamitrinib. Astrocytes were treated with various concentrations of Thap in the presence of 0, 2.5, 5, or 10 μM gamitrinib and the cell viability was analyzed by MTT assay. Data are from three independent experiments. **(C)** TRAP1 expression in astrocytes. TRAP1 and cyclophilin D (Cyp-D) expression in astrocytes isolated from mouse brain was compared with cancer cell lines by western blotting. **(D)** Tumor xenograft experiment. Subcutaneous 22Rv1 xenografts were established as described in Materials and Methods. At the end of the experiment, final tumor volumes were plotted (right). We used five mice per group and two tumors per animal. **(E)** Analysis of CHOP expression in liver and tumor. Liver and tumor samples from three randomly selected mice for each treatment (total 12 mice) were analyzed by western blotting (left). After normalization of CHOP band intensities with β-actin, relative CHOP intensities were calculated (right). **(F)** Schematic diagram of the mitochondria-initiated stress signal. Chemical inhibitors are indicated in red. **(B)**, **(D)**, and **(E)** are the mean ± SEM. ***, *p =* 0.0003; *, *p* = 0.039; #, *p* > 0.1.

The mitochondrial Hsp90 pool is dramatically elevated in many cancer cells to cope with various stresses, but expression is very low or undetectable in most normal tissues except brain and testis [7,8,10,51,52]. To test whether mitochondrial Hsp90-regulated interorganelle calcium signaling is functional in normal cells, we examined primary astrocytes from mouse brain, where Hsp90 expression in mitochondria is higher than in other tissues [7]. Gamitrinib did not affect CHOP induction and eIF2α phosphorylation (Figure 6A), whereas Thap increased CHOP expression in astrocytes (Additional file 1: Figure S6A). Gamitrinib treatment in combination with Thap did not sensitize astrocytes (Figure 6B), possibly due to very low expression of both TRAP1 and Cyp-D in astrocytes compared to cancer cells (Figure 6C). Collectively, gamitrinib does not affect the cell death threshold in astrocytes, probably due to the limited contribution of the chaperones to PTP opening in normal cells; this is in stark contrast with data from cancer cells (Figure 4B-D). Next, the gamitrinib and Thap combination was further examined using a xenograft of relapsed prostate cancer cells (22Rv1) [53], to test whether the cancer cell-specific lowering of the cell death threshold occurs *in vivo*. Because Thap has been reported to be highly toxic *in vivo*[54], we administered a very low dose of the drug. Suboptimal individual doses of Thap and gamitrinib did not result in significant inhibition of tumor growth, whereas combined treatment inhibited tumor growth (Figure 6D) without remarkable histological abnormalities and body weight changes (Additional file 1: Figure S6B and C). Individual treatment with either gamitrinib or Thap slightly elevated CHOP expression, whereas combined treatment further elevated CHOP expression synergistically in cancer cells, but not in the brain or liver (Figure 6E; Additional file 1: Figure S6D). Therefore, similar to the *in vitro* data, mitochondrial Hsp90 inhibition lowers the cell death threshold of cancer cells to Thap treatment *in vivo*.

## Discussion

Mitochondria are integrators of various cellular stress signals that eventually make life-or-death decisions. We show here that mitochondria can also produce calcium-mediated stress signals and propagate them to neighboring organelles. For calcium signaling, interplay between the permeability transition pore (PTP) in mitochondria and ryanodine receptor (RyR) in ER was essential, and the mitochondrial Hsp90 pool negatively modulates signal commencement in cancer cells to protect them from cellular stresses.

TRAP1 knockdown by siRNA showed a similar phenotype to simultaneous inactivation of both Hsp90 and TRAP1 by gamitrinib. Considering functional overlap between Hsp90 and TRAP1 in the regulation of PTP in cancer cells [7,55], the lack of functional compensation by the mitochondrial Hsp90 is quite unexpected, and may suggest different protein interaction networks between Hsp90 and TRAP1, or alternatively, that TRAP1 functionally dominates over Hsp90 in cancer mitochondria. There is growing consensus that mitochondrial Hsp90 and TRAP1 play important roles in neoplastic progression by modulating a variety of mitochondrial pathways: metabolic reprogramming, mitochondrial dynamics, reactive oxygen species, autophagy, and cell death [55,56]. Thus, to clearly address their roles in mitochondrial homeostasis and tumorigenesis, the relative contribution of the chaperones to the mitochondrial signal pathways and the functional relationship between them should first be discovered.

Calcium, in such stress signaling, does not merely mediate mitochondria-ER communication, but is also critical for PTP and RyR calcium channel opening. Calcium released through one of the channels can trigger the opening of the other through calcium-induced calcium release (CICR) [19,32], which can eventually amplify signals even with minute perturbation of mitochondrial chaperone functions (Figure 6F). Interestingly, IP_3_Rs, major ER calcium channels allegedly requiring the ligand IP_3_ as well as calcium for CICR [44], are not involved in mitochondria-initiated calcium signaling, which further argues that the mitochondria-initiated pathway occurs through genuine CICR that is solely dependent on discharged calcium.

Interplay and subsequent signal amplification between the calcium channels are crucial to signaling, as inhibition of either the PTP or RyR blocked calcium signaling. The unknown mechanism of CHOP induction after inactivation of mitochondrial Hsp90s in previous reports [4,38-40] can be explained by this interplay. Furthermore, local calcium increases, seen as calcium hot-spots after low-dose gamitrinib treatment (Fluo-4 staining in Figure 4B), seem to be the consequence of the calcium channel interplay, which is sufficient to provoke global mitochondrial membrane potential reduction and UPR^ER^ induction. Without a large increase in cytoplasmic calcium concentration, this is sufficient to propagate the stress response, probably due to the closely apposed architecture of the mitochondria and ER [29-31]. Thus, mitochondria-initiated calcium signaling might be further supported by the physical interconnection between mitochondria and the ER, which forms a specialized microdomain of transient calcium [28,57] and can facilitate reciprocal PTP and RyR activation via CICR.

Target-centered anticancer drugs often show limited efficacies, poor safety, and resistance profiles due to complicated signaling networks in many cancer cells [58,59]. Multicomponent and system-oriented therapeutics development approaches could provide a solution [60,61]. The target proteins of gamitrinib and Thap have fundamentally different functions in distinct organelles [34,62,63]. When combined, their anticancer activities were enhanced and non-toxic doses of the drugs were sufficient *in vitro* and *in vivo* to kill cancer, but not normal cells, through calcium-mediated coordination of compartmentalized signaling networks and synergistic elevation of CHOP expression. These pharmacological data further support the function of mitochondrial Hsp90s as important regulators of interorganelle crosstalk, increasing the stress threshold, and identify these proteins as drug targets for the development of novel combination cancer therapy. Thus, we believe that mitochondrial Hsp90 inhibitors require further system-oriented investigation to facilitate the development of an effective and better multicomponent anticancer regimen by combining antitumor drugs or even non-antitumor drugs capable of inducing organelle stress.

## Conclusions

Mitochondria-initiated and calcium-mediated propagation of the stress signal plays an important role in coordinating ER and mitochondrial stress responses, and is implicated in lowering the cell death threshold in cancer cells. Therefore, targeting the coordinated calcium stress signaling pathway often suppressed in cancer cells might be a feasible and effective strategy for the rational development of cancer therapeutics.

## Materials and methods

### Cells and culture condition

HeLa, MDA-MB-231, and NCI-H460 cells were purchased from the Korean Cell Line Bank and 22Rv1 from the American Type Culture Collection. Cell lines were maintained as recommended by supplier. Cells were cultured in DMEM or RPMI medium (Lonza) containing 10% fetal bovine serum (FBS; GIBCO) and 1% penicillin/streptomycin (GIBCO) at 37°C in a 5% CO_2_ humidified atmosphere.

### Chemicals, plasmids and antibodies

Gamitrinib conjugated with triphenylphosphonium was prepared as described previously [33]. MitoTracker, Fura-2-AM, and tetramethylrhodamine methyl ester (TMRM) were purchased from Molecular Probes, Ryanodine was from Santa Cruz Biotechnology. Mn(III) tetrakis(1-methyl-4-pyridyl) porphyrin (MnTMPyP) was from Calbiochem. 1,2-bis(o-aminophenoxy) ethane-N,N,N’,N’-tetraacetic acid acetoxymethyl ester (BAPTA), cyclosporine A (CsA), carbonyl cyanide 4-(trifluoromethoxy) phenylhydrazone (FCCP), tetracaine, and thapsigargin (Thap), and N-acetylcysteine (NAC) and all other chemicals, were from Sigma.

Anti-CEBP homologous protein (CHOP) antibodies were obtained from Cell Signaling; anti-RyR, anti-IP_3_R, anti-eIF2α and anti-cytochrome *c* antibodies from Santa Cruz Biotechnology; anti-cyclopholin D from Calbiochem; anti-eIF2α[pS52] from Invitrogen; anti-β-actin from MP Biomedicals; and anti-TRAP1 from BD Biosciences.

### Astrocyte preparation

Primary cultures of astrocytes were prepared as previously described [64]. Briefly, the mouse brain cortex, after removing the meninges, was dissected and dissociated with moderate pipetting. Cells were plated on 100-mm dishes coated with 10 μg/ml poly-D-lysine (Sigma) and grown to confluence in DMEM supplemented with 10% FBS, 10% horse serum (GIBCO), 100 units/ml penicillin, and 100 μg/ml streptomycin at 37°C in a 5% CO_2_ humidified atmosphere. Afterward, astrocytes were trypsinized and plated on 6-well plates coated with poly-D-lysine to administer drugs.

### siRNA treatment

Small interfering RNAs (siRNA) against TRAP1, RyR2, IP_3_R, and CHOP were synthesized by Genolution (Korea) as follows:

RyR2-#1, 5′-AAGTGGTTCTGCAGTGCACCG; RyR2-#2, 5′-AAGTACGAGTTGGAGATGACC; TRAP1-#1, 5′- AAACATGAGTTCCAGGCCGAG; TRAP1-#2, 5′- CCCGGTCCCTGTACTCAGAAA; IP_3_R1-#1, 5′-GAGAATTTCCTTGTAGACATCTGCA; IP_3_R1-#2, 5′-GGCCTGAGAGTTACGTGGCAGAAAT; IP_3_R2, 5′-GAGAAGGCTCGATGCTGAGACTTGA; IP_3_R3, 5′-CCGAGATGACAAGAAGAACAAGTTT; CHOP-#1, 5′-AGAACCAGCAGAGGTCACAA; CHOP-#2, 5′-AAGAGAATGAACGGCTCAAGC; control, 5′-ACUCUAUCUGCACGCUGAC. Cells were cultured on 6-well plates at 50–75% confluence, transfected with 20 nM siRNA mixed with G-Fectin (Genolution) for 48 hours, and then analyzed or treated with drugs.

### Analysis of cell viability and apoptosis induction

Cells (5 × 10^3^ cells/well) were cultured in 96-well plates overnight and treated with gamitrinib and Thap alone or in combination for 24 hours. To determine cell viability, cells were exposed to 3 (4,5-dimethyl-thyzoyl-2-yl)2,5 diphenyltetrazolium bromide (MTT), and crystalized formazan was quantified by measuring the absorbance at 595 nm with an Infinity M200 microplate reader (TECAN). Absorbance data were compared with that of vehicle control and expressed as percent viability. Alternatively, after treatment with drugs, DNA content (propidium iodide, red fluorescence) and caspase activation (DEVDase activity, green fluorescence) of the cells were analyzed using the CaspaTag *in situ* apoptosis detection kit (Millipore). Labeled cells were analyzed using the FACS Calibur™ system (BD Biosciences). Data were processed using FlowJo software (TreeStar).

### CHOP reporter assay

To generate a CHOP reporter stable cell line, PC 3 cells were co-transfected with 8 μg of a promoter construct (*CHOP*::*GFP*) [50] obtained from Addgene (Addgene plasmid 21898) and 800 ng of puromycin linearized selection marker (Clontech) using Lipofectamin (Invitrogen) per manufacturer’s instructions. Transfected PC3 cells were cultured in RPMI (Lonza) with 1 μg/ml puromycin (Clontech) for 3 weeks and colonies were picked using cloning cylinders. GFP expression was monitored in the IncuCyte™ imaging system (Essen Bioscience) at an excitation wavelength of 450–490 nm and an emission of 500–530 nm, and analyzed by Image J software (National Institutes of Health).

### Live cell imaging for intracellular calcium

HeLa cells were incubated with 5 μM Fura-2-AM for 30 min at 37°C and 5% CO_2_. After washing with Hank’s Buffer, the cells were incubated with calcium-free Locke’s solution (154 mM NaCl, 5.6 mM KCl, 3.2 mM MgCl_2_, 5 mM HEPES, 10 mM glucose, 0.2 mM EGTA; pH 7.4). Fluorescence changes were monitored every 5 minutes using an IX81 ZDC microscope (Olympus) at an emission wavelength of 510 nm with dual excitation at 340 nm and 380 nm. Images of the 340/380 fluorescence ratio were generated and analyzed by the Xcellence software package (Olympus).

### Imaging D1ER and mtCameleon

Fluorescence resonance energy transfer (FRET) measurements were performed using an FV1000 laser confocal scanning microsope (Olympus) with a FRET module and a UPLSAPO 100× oil immersion objective with a 1.40 numerical aperture. HeLa cells were seeded on a Lab Tek II slide chamber at 40–80% confluency in DMEM (Lonza) supplemented with 10% FBS and 1% penicillin/streptomycin at 37°C and 5% CO_2_. D1ER or mtCameleon constructs (kind gifts from Dr. R.Y. Tsien, University of San Diego) [37] were transfected into HeLa cells using the Lipofectamine transfection reagent (Invitrogen) per manufacturer’s instructions. Cells were imaged at 24 or 48 hours after transfection. All analyses were performed under the same conditions. D1ER and mtCameleon, containing FRET donor (CFP) and acceptor (citrine) components, were excited with a 440-nm diode laser source; the emitted fluorescence bands were separated by a grating and detected by photomultiplier tubes in the CFP channel (480 nm) and FRET channel (535 nm). The FRET ratio (R_FRET_) was calculated as described previously [65] from confocal images using FV10-ASW 3.1 software (Olympus) by pixel-by-pixel quantification of fluorescence intensity: R_FRET_ = I_FRET_/I_CFP_ , where I_FRET_ and I_CFP_ represent the fluorescence intensities from the FRET and CFP channels, respectively. The FRET ratio (relative units) was plotted after comparing R_FRET_ values.

### RNA extraction and reverse transcript-PCR

Total RNA was prepared from cells suspended in cold PBS using the RNeasy mini kit (QIAGEN), and cDNA was synthesized using the ProtoScript® First Strand cDNA Synthesis Kit (New England Biolabs) using an oligo(dT) primer. The PCR reaction was performed in a Mastercycler PCR machine (Eppendorf) with the following sets of oligonucleotide primers: glyceraldehyde phosphate dehydrogenase (GAPDH), 5′-CGGGAAGCTTGTCATCAATGG-3′ and 5′-GGCAGTGATGGCATGGACTG-3′; CHOP, 5′- CTTTCTCCTTCGGGACACTG-3′ and 5′-AGCCGTTCATTCTCTTCAGC-3′; TRAP1, 5′- ATGGCGCGCGAGCTGCGG-3′ and 5′-CAGTCGTCCTGCCTGCAA-3′; X-box binding protein 1 (XBP1), 5′-CCTTGTAGTTGAGAACCAGG-3′ and 5′-GGGGCTTGGTATATATGTGG-3′.

### Xenograft tumor models

All experiments involving animals were approved by UNIST (IACUC-12-003-A). 22Rv1 (7 × 10^6^) cells suspended in sterile PBS (200 μl) were injected subcutaneously into both flanks of 6-week-old BALB/c nu/nu male mice (Japan SLC Inc.) and allowed to grow to an average volume of approximately 100 mm^3^. Animals were randomly divided into four groups (two tumors/mouse, five mice/group). Gamitrinib or vehicle (DMSO) dissolved in 20% Cremophor EL (Sigma) in PBS was injected intraperitoneally, and Thap dissolved in 0.9% NaCl in PBS intravenously. The mice were administered 10 mg/kg gamitrinib and 0.2 mg/kg Thap twice a week. Tumors were measured daily with a caliper, and tumor volume was calculated using the formula: V = 1/2 × (width)^2^ × length. At the end of experiment, animals were euthanized, and organs including brain, heart, kidney, liver, lung, spleen, and tumor were collected for histology or western blotting. For histological analysis, harvested organs were fixed in 10% formalin and embedded in paraffin. Sections (5 μm) were placed on high-adhesive slides, stained with H&E, and scanned using the Dotslide system (Olympus) with 10× magnification. For western blot analysis, tissue samples were lysed in RIPA buffer (50 mM Tris, pH 8.0, 150 mM NaCl, 1% NP-40, and 0.25% N-deoxycholate) containing protease inhibitor and phosphatase inhibitor cocktails (Calbiochem) using a homogenizer (IKA).

### Statistical analysis of data

All MTT experiments were duplicated and repeated independently at least three times. Statistical analyses were performed using the software program Prism 5.0 (GraphPad). In an unpaired *t*-test, *p* < 0.05 was considered significant.

## Abbreviations

BAPTA: 1,2-bis(o-aminophenoxy)ethane-N,N,N’,N’-tetraacetic acid acetoxymethyl ester; CHOP: CEBP homologous protein; CICR: Calcium induced calcium release; CsA: Cyclosporine A; Cyp-D: Cyclophilin D; Δ*Ψ*m: Mitochondrial membrane potential; eIF2α: Eukaryotic translation initiation factor 2α; ER: Ndoplasmic reticulum; FCCP: Carbonyl cyanide 4-(trifluoromethoxy) phenylhydrazone; Hsp90: Heat shock protein 90; IP_3_R: Inositol 1,4,5-trisphosphate receptors; MOMP: Mitochondrial outer membrane permeabilization; MTT: 3(4,5-dimethyl-thyzoyl-2-yl)2,5 diphenyltetrazolium bromide; PTP: Permeability transition pore; 17AAG: 17-allylamino-17-demethoxygeldanamycin; RyR: Ryanodine receptor; Thap: Thapsigargin; TMRM: Tetramethylrhodamine methyl ester; TRAP1: Tumor necrosis factor receptor-associated protein 1; UPR: Unfolded protein response; XBP1: X-box binding protein 1.

## Competing interests

The authors declare that they have no competing interests.

## Authors’ contributions

BHK and HKP designed experiments, analyzed data, wrote the manuscript. HKP conducted most of the experiments. JEL analyzed cell viability and apoptosis induction after drug treatment. JL participated in CHOP reporter assay and animal experiments. All authors reviewed and approved the final manuscript.

## Supplementary Material

Additional file 1: Figure S1 Effect of 17AAG on calcium concentration in the cytoplasm/mitochondria and gamitrinib normal cell effect. **Figure S2.** Inhibition of mitochondrial Hsp90s activates ER stress sensors. **Figure S3.** IP_3_ receptors and lysophosphatidic acid (LPA)-induced calcium flux. **Figure S4.** Sensitization of cancer cells to thapsigargin by mitochondrial Hsp90 inhibition. **Figure S6.** Effect of combination drug treatment on normal tissues. Click here for file
