# Supplementary material for: Mitochondrial Hsp90s suppress calcium-mediated stress signals propagating from mitochondria to the ER in cancer cells
Source: Mol Cancer. 2014 Jun 12;13:148. doi: 10.1186/1476-4598-13-148 (PMC4070406; doi:10.1186/1476-4598-13-148)
Supplement: Additional file 1: Figure S1 — Effect of 17AAG on calcium concentration in the cytoplasm/mitochondria and gamitrinib normal cell effect. Figure S2. Inhibition of mitochondrial Hsp90s activates ER stress sensors. Figure S3. IP3 receptors and lysophosphatidic acid (LPA)-induced calcium flux. Figure S4. Sensitization of cancer cells to thapsigargin by mitochondrial Hsp90 inhibition. Figure S6. Effect of combination drug treatment on normal tissues. [file 1476-4598-13-148-S1.pdf]

**Additional file 1**

# Mitochondrial Hsp90s suppress calcium signals propagating from mitochondria to the ER in cancer cells

Hye-Kyung Park, Ji-Eun Lee, Jaehwa Lim, and Byoung Heon Kang

Department of Biological Sciences, School of Life Sciences, UNIST, Ulsan, 689-798, Korea

## Supplementary Figure Legends

Figure S1. Drug effect on mitochondria.

(A) No elevation of cytosolic calcium on 17AAG treatment. Fura-2 loaded HeLa cells were treated with a non-targeted Hsp90 inhibitor, 17AAG, and analyzed using a fluorescence microscope.

(B) Gamitrinib effect on MCF10A. TMRM loaded MCF10A cells were treated with 30 μM gamitrinib as indicated and analyzed by confocal microscope (left). Quantitation of the images (right). Data are mean ± SEM from 40 ROIs. #, not significant; *, $p<0.0001$.

(C) Organelle specific effect of FCCP and Thap. TMRM loaded HeLa cells were treated with 10 μM FCCP or 10 μM Thap for 30 mins and analyzed by confocal microscope (left). Quantitation of the confocal microscope images (middle). Data are mean ± SEM from 30 ROIs. #, not significant; *, $p<0.0001$. HeLa cells were treated with the drugs for 2 hours and analyzed by western blotting (right).

(D) Effect of 17AAG on the mitochondrial calcium store. mtCameleon was transiently expressed in HeLa cells. FRET images were acquired after 30 μM 17AAG treatment at the indicated time intervals (left) and the FRET ratio was calculated and plotted (right). Data are the mean ± SEM calculated from 30 cells in two independent experiments. Bar, 10 μm. R.U., relative units.

Figure S2. Inhibition of mitochondrial Hsp90s activates ER stress sensors

(A) XBP1 splicing by silencing TRAP1. HeLa cells were treated with two different TRAP1 siRNAs for 24 hours. mRNA expression levels of XBP1 splicing variants, TRAP1, and GAPDH were analyzed by RT-PCR.

(B) eIF2α phosphorylation upon TRAP1 silencing. After treatment of HeLa cells with two different TRAP1 siRNAs, the expression levels of eIF2α, phospho-eIF2α (p-eIF2α), TRAP1, and β-actin were analyzed by western blotting.

(C) XBP1 splicing upon mitochondrial Hsp90 inhibition. After treatment with 30 μM gamitrinib for 2 hours, mRNA expression levels of XBP1 splicing variants, TRAP1, and GAPDH mRNAs were analyzed by RT-PCR.

(D) Analysis of CHOP mRNA. HeLa cells were treated with 30 μM gamitrinib or TRAP1 siRNAs as indicated. CHOP and GAPDH mRNAs were analyzed by RT-PCR.

Figure S3. $IP_3$ receptors and lysophosphatidic acid (LPA)-induced calcium flux.

(A) Silencing of $IP_3R$ isotypes. HeLa cells were treated with $IP_3R1$-, $IP_3R2$-, and $IP_3R3$-specific siRNAs, and analyzed by Western blotting.

(B) $IP_3R1$ silencing blocked LPA-induced calcium flux. HeLa cells were treated with $IP_3R1$ siRNA and labeled with Fura-2. After LPA treatment, cytoplasmic calcium flux was monitored as in Fig S1A. Data are the mean ± SEM calculated from 20 ROIs in two independent experiments.

Figure S4. Sensitization of cancer cells to thapsigargin by mitochondrial Hsp90 inhibition.

Knockdown of TRAP1 by siRNA. After silencing of TRAP1 in HeLa and 22Rv1 cells with TRAP1-#1 siRNA, cells were treated with various concentrations of Thap for 24 hours and analyzed by the MTT assay. Data are from three independent duplicate experiments; data are given in terms of mean ± SEM.

Figure S5. Death receptor 5 (DR5) and reactive oxygen species (ROS) in the drug

combination

(A) DR5 expression and caspase-8 activation. 22Rv1 cells were treated with 2.5 μM gamitrinib and 0.06 μM Thap as indicated for 24 hours, then analyzed by reverse transcription (RT)-PCR and western blotting.

(B) ROS do not affect gamitrinib-induced calcium release. Fura-2 loaded 22Rv1 cells were incubated with 30 mM N-acetylcysteine (NAC) or 30 μM manganese (III) tetrakis (1-methyl-4-pyridyl) porphyrin pentachloride (MnTMPyP) for 1 hour and then treated with 30 μM gamitrinib for 2 hours before being analyzed by the fluorescence microscope.

(C) ROS scavenger effect on CHOP induction. 22Rv1 cells were treated with 20 μM gamitrinib in the presence or absence of 20 μM MnTMPyP for 8 hours and analyzed by western blotting (left). Similarly, the cells were treated with 0.5 mM ascorbic acid, 0.5 mM 2,2,6,6 tetramethylpiperidine 1-oxyl (TEMPO), and 20 μM pyrollidine dithiocarbamate (PDTC) for 8 hours and analyzed by western blotting (right).

Figure S6. Effect of combination drug treatment on normal tissues

(A) Gamitrinib and Thap treatment in astrocytes. Astrocytes were treated with 10 μM Thap or 30 μM gamitrinib for 4 hours and 1 μM Thap or 2.5 μM gamitrinib for 24 hours, and analyzed by western blotting.

(B) Hematoxylin and eosin staining. The animals were sacrificed at the end of the experiment, and organs were collected, fixed, stained, and analyzed under a light microscope.

(C) Mouse body weight change. Prior to sacrificing the animals in the xenograft experiment, their body weights were measured. Data are mean ± SEM.

(D) Analysis of CHOP expression in mouse brain. Brain samples from sacrificed mice

(total 8 mice) were analyzed by western blotting.

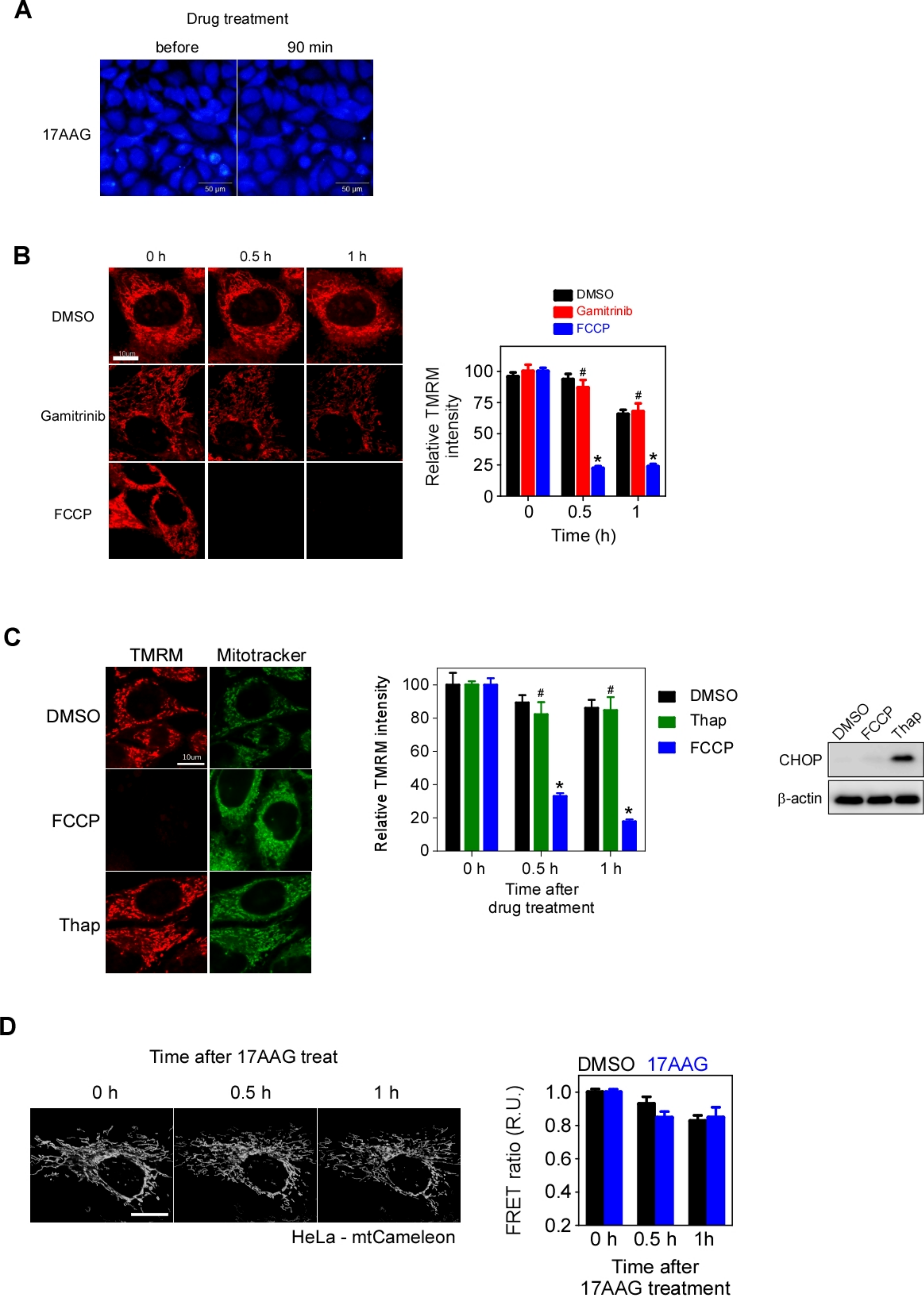

A
Drug treatment
before
90 min
17AAG
50 μm
50 μm
B
0 h
0.5 h
1 h
DMSO
Gamitrinib
FCCP
10um
DMSO
Gamitrinib
FCCP
Relative TMRM intensity
100
75
50
25
0
0
0.5
1
Time (h)
#
#
*
*
C
TMRM
Mitotracker
DMSO
FCCP
Thap
10um
Relative TMRM intensity
100
80
60
40
20
0
0 h
0.5 h
1 h
Time after drug treatment
DMSO
Thap
FCCP
DMSO
FCCP
Thap
CHOP
β-actin
D
Time after 17AAG treat
0 h
0.5 h
1 h
HeLa - mtCameleon
DMSO
17AAG
FRET ratio (R.U.)
1.0
0.8
0.6
0.4
0.2
0 h
0.5 h
1h
Time after 17AAG treatment


Figure S1

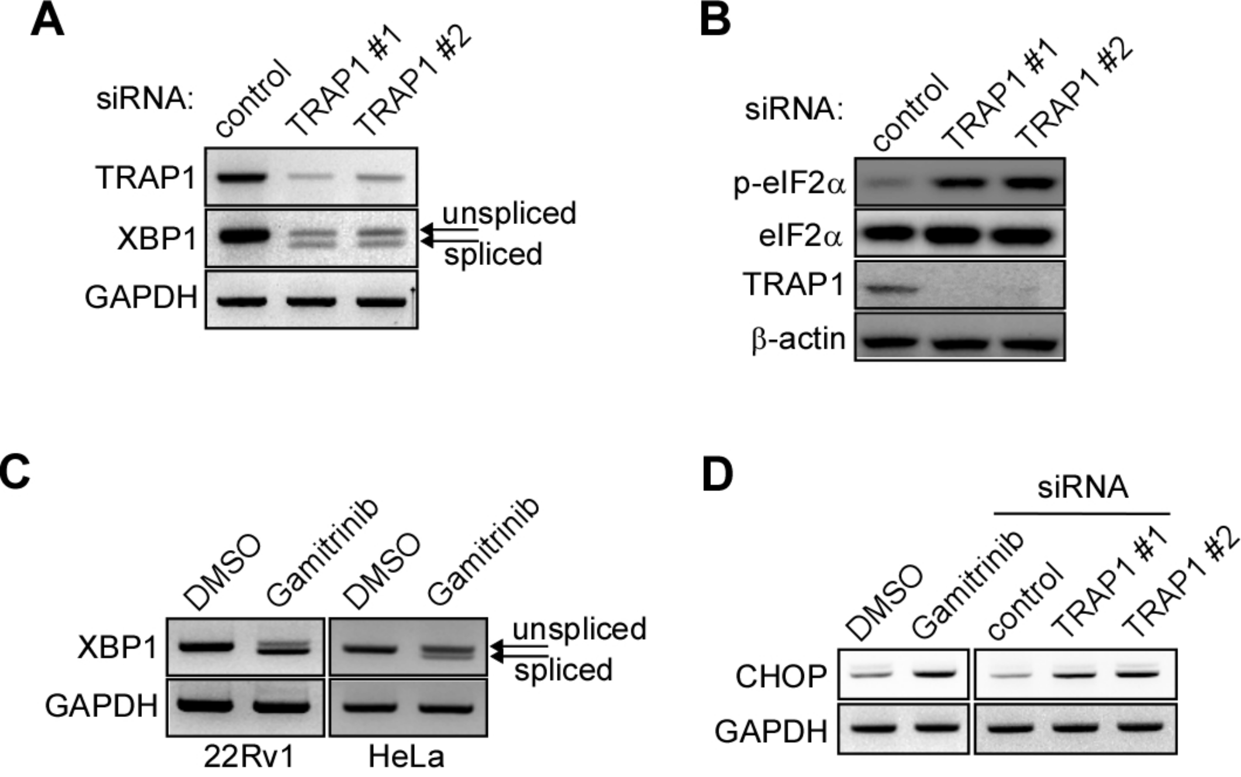
A
siRNA:
control
TRAP1 #1
TRAP1 #2
TRAP1
XBP1
GAPDH
unspliced
spliced
B
siRNA:
control
TRAP1 #1
TRAP1 #2
p-eIF2α
eIF2α
TRAP1
β-actin
C
DMSO
Gamitrinib
DMSO
Gamitrinib
XBP1
GAPDH
unspliced
spliced
22Rv1
HeLa
D
siRNA
DMSO
Gamitrinib
control
TRAP1 #1
TRAP1 #2
CHOP
GAPDH

Figure S2

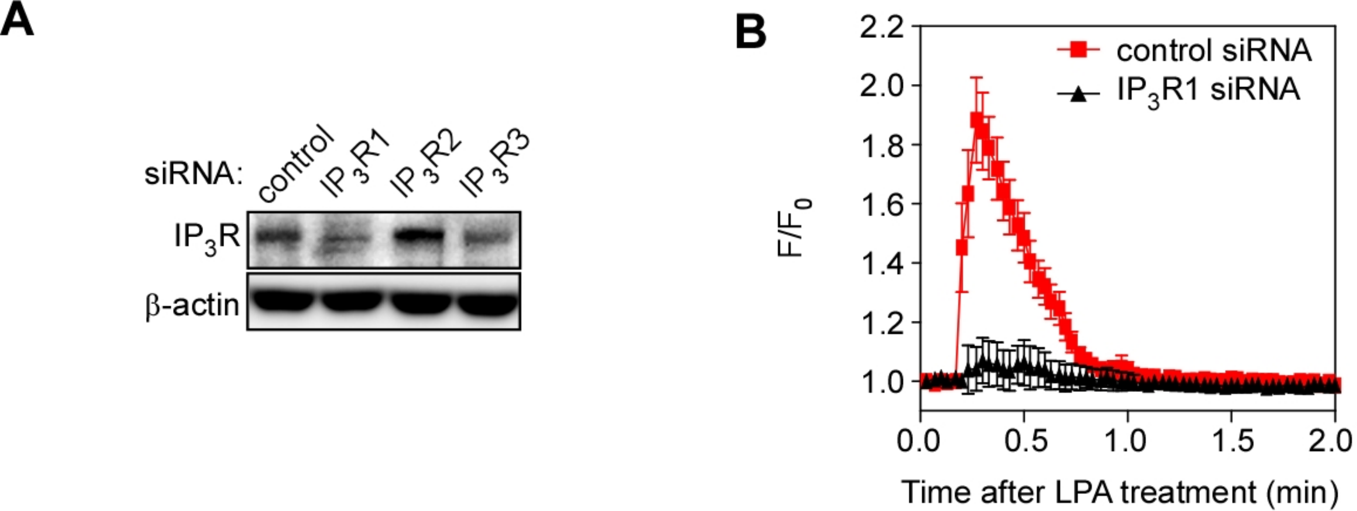
A
siRNA:
control
IP3R1
IP3R2
IP3R3
IP3R
β-actin
B
control siRNA
IP3R1 siRNA
F/F0
2.2
2.0
1.8
1.6
1.4
1.2
1.0
0.0
0.5
1.0
1.5
2.0
Time after LPA treatment (min)

Figure S3

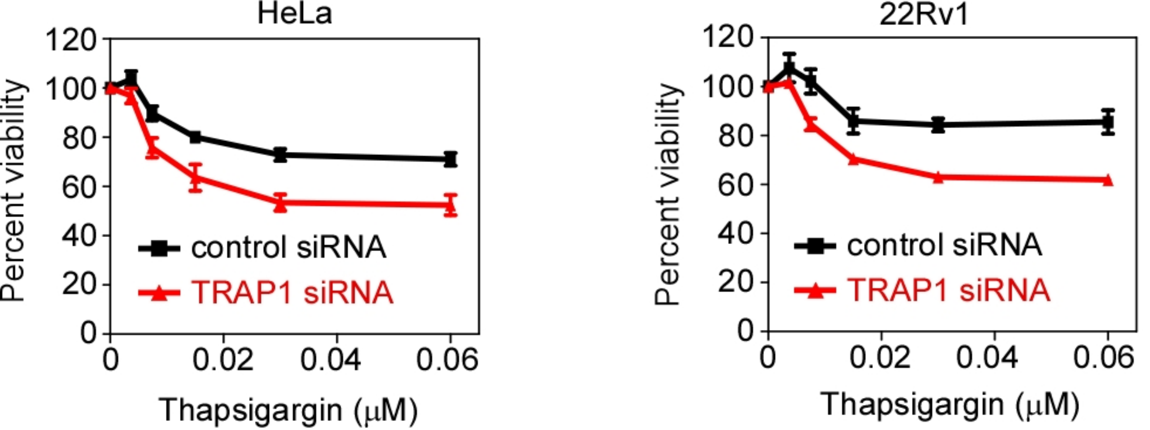

HeLa
Percent viability
120
100
80
60
40
20
0
0
0.02
0.04
0.06
Thapsigargin (μM)
control siRNA
TRAP1 siRNA
22Rv1
Percent viability
120
100
80
60
40
20
0
0
0.02
0.04
0.06
Thapsigargin (μM)
control siRNA
TRAP1 siRNA


Figure S4

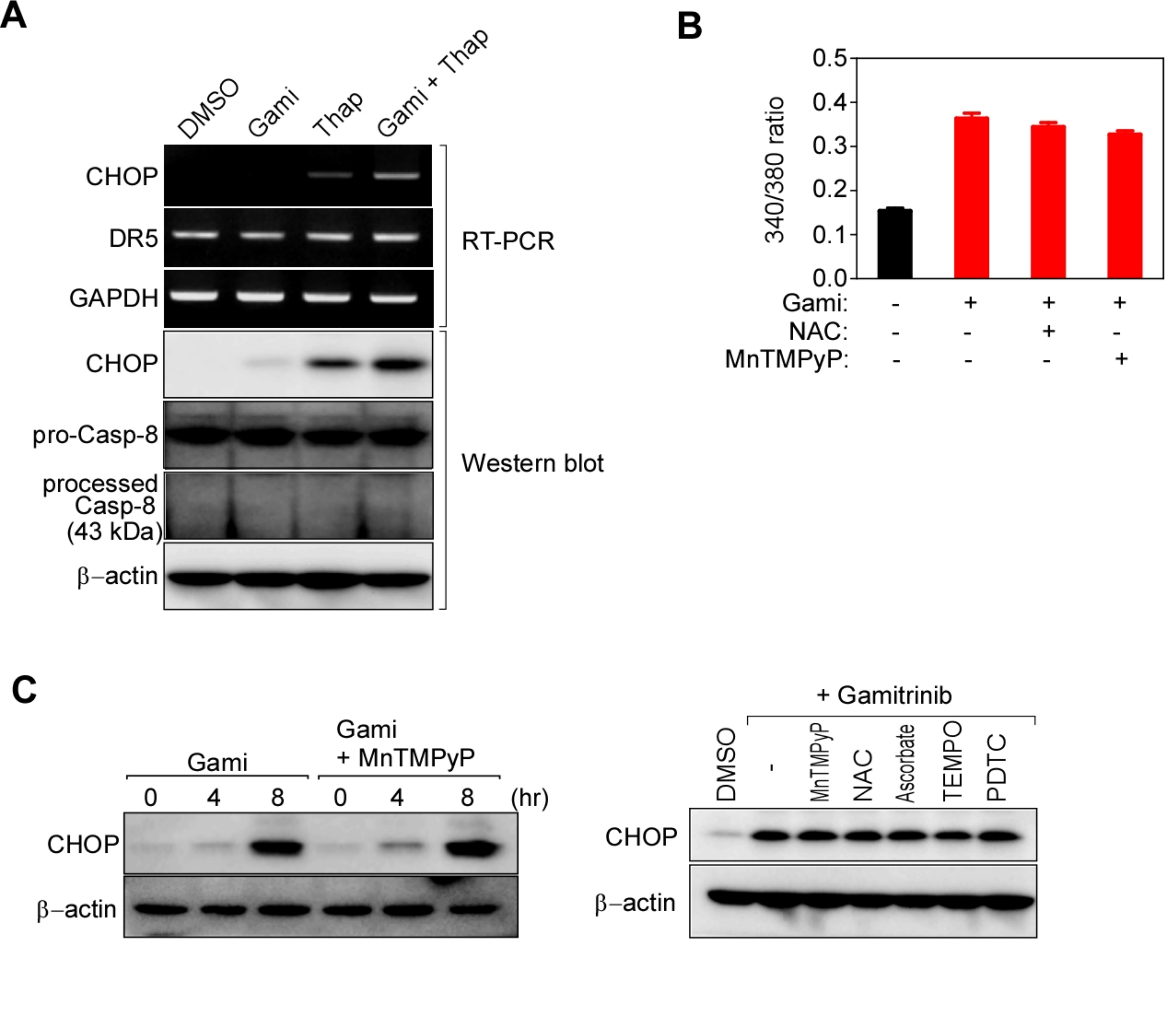
A
DMSO
Gami
Thap
Gami + Thap
CHOP
DR5
GAPDH
RT-PCR
CHOP
pro-Casp-8
processed Casp-8 (43 kDa)
β−actin
Western blot
B
340/380 ratio
0.5
0.4
0.3
0.2
0.1
0.0
Gami: - + + +
NAC: - - + -
MnTMPyP: - - - +
C
Gami
Gami + MnTMPyP
0 4 8 0 4 8 (hr)
CHOP
β−actin
+ Gamitrinib
DMSO
-
MnTMPyP
NAC
Ascorbate
TEMPO
PDTC
CHOP
β−actin

Figure S5

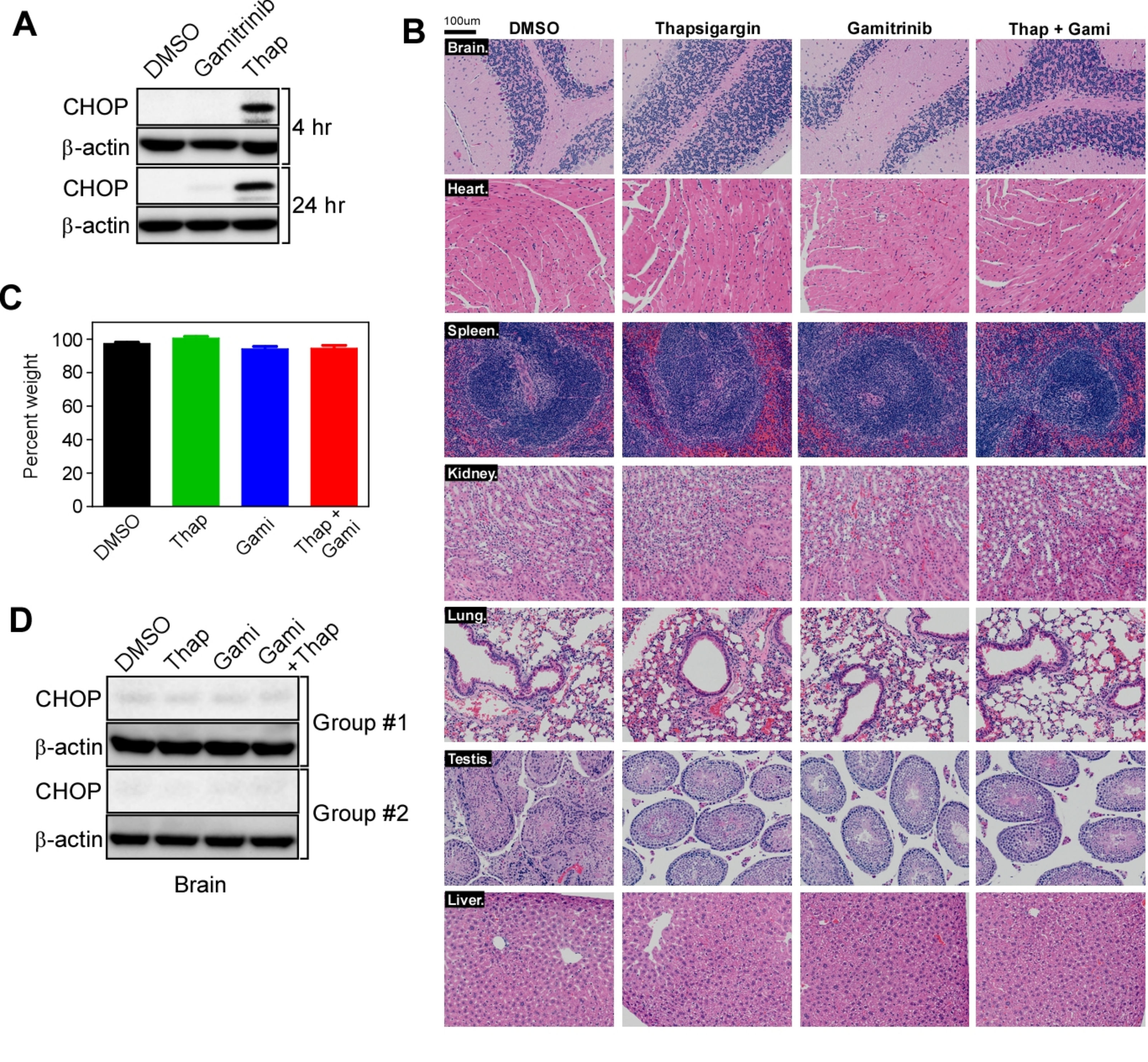

A
DMSO
Gamitrinib
Thap
CHOP
β-actin
4 hr
CHOP
β-actin
24 hr
B
100um
DMSO
Thapsigargin
Gamitrinib
Thap + Gami
Brain.
Heart.
Spleen.
Kidney.
Lung.
Testis.
Liver.
C
Percent weight
100
80
60
40
20
0
DMSO
Thap
Gami
Thap + Gami
D
DMSO
Thap
Gami
Gami +Thap
CHOP
β-actin
Group #1
CHOP
β-actin
Group #2
Brain


Figure S6
